# Prenatal Cadmium Exposure Is Negatively Associated With Adiposity in Girls Not Boys During Adolescence

**DOI:** 10.3389/fpubh.2019.00061

**Published:** 2019-04-12

**Authors:** Meghan Moynihan, Martha Maria Telléz-Rojo, Justin Colacino, Andrew Jones, Peter X. K. Song, Alejandra Cantoral, Adriana Mercado-García, Karen E. Peterson

**Affiliations:** ^1^Department of Nutritional Sciences, School of Public Health, University of Michigan, Ann Arbor, MI, United States; ^2^Center for Research on Nutrition and Health, National Institute of Public Health, Cuernavaca, Mexico; ^3^Department of Environmental Health Sciences, School of Public Health, University of Michigan, Ann Arbor, MI, United States; ^4^Center for Human Growth and Development, University of Michigan, Ann Arbor, MI, United States; ^5^Department of Biostatistics, School of Public Health, University of Michigan, Ann Arbor, MI, United States

**Keywords:** adiposity, adolescence, cadmium, prenatal, sex-dependent

## Abstract

**Introduction:** Cadmium is a pervasive toxic metal that remains a public health concern and exposure in early life has been associated with growth deficits in infancy and childhood. Growth during adolescence also may be sensitive to effects of cadmium exposure, given the changes in distribution of lean and adipose tissue that vary by sex during puberty. This study examines whether prenatal and concurrent cadmium exposures are associated with adiposity measures at ages 8–15 years in a well-characterized birth cohort.

**Methods:** The sample included 185 participants from the ELEMENT birth cohorts in Mexico City with complete data on urinary cadmium exposures, anthropometry and covariates [child age and sex, household socioeconomic status, and maternal smoking history and body mass index (BMI)]. Maternal third trimester and adolescent urines were analyzed for cadmium using an Inductively Coupled Plasma Mass Spectrometer. Trained personnel obtained anthropometry including height, weight, waist circumference and subscapular, suprailiac, and triceps skinfold thickness. BMI z-scores for age and sex were calculated using the World Health Organization's reference standard. Linear regression models were used to estimate the association of prenatal and concurrent urinary cadmium levels with adolescent anthropometry, adjusting for covariates.

**Results:** Among 87 males and 98 females, median age was 10 years (IQR 9 –11 years). Pregnant women and children had median urinary cadmium concentrations of 0.19 μg/L (IQR 0.12– 0.27 μg/L) and 0.14 μg/L (IQR 0.11– 0.18 μg/L), respectively. Regression models showed inverse relationships between prenatal cadmium exposure and adolescent adiposity. An IQR increase in prenatal cadmium was associated with percent decreases in BMI z-score (−27%, *p* = 0.01), waist circumference (−3%, *p* = 0.01), and subscapular (−11%, *p* = 0.01), suprailiac (−11%, *p* = 0.02), and triceps (−8%, *p* < 0.01) skinfold thickness. When stratified by sex, these relationships remained statistically significant in females but not males.

**Conclusions:** Prenatal cadmium exposure was negatively associated with measures of both abdominal and peripheral adiposity in girls, but not in boys. These results emphasize the sex-dependent effects of *in utero* cadmium exposure on adiposity in adolescence.

## Introduction

Cadmium is a pervasive toxic metal with two main routes of human exposure: inhalation of cigarette smoke and ingestion of foods with high levels of cadmium, such as leafy vegetables, potatoes, grains, peanuts, soybeans, and sunflower seeds (1). Although largely sourced from healthy foods—vegetables and whole grains (2)—cadmium exposure is associated with several health outcomes even at low exposure levels (3). Prenatal exposure is a public health concern for children, where sensitivities to cadmium can be pronounced and have lasting health effects (4, 5).

Several prospective studies have related prenatal cadmium exposure to growth deficits in infancy and early childhood (6–10). Cross-sectional studies in adolescents have shown inverse relationships of cadmium exposure with abdominal and subcutaneous fat (11, 12) and obesity (13, 14). One report examining the effects of both prenatal and childhood cadmium exposure on growth in a rural Bangladesh population (10) found the strongest inverse relationship between concurrent exposure and height at 5 years of age. These findings suggest that physical growth during both prenatal and postnatal periods may be sensitive to effects of cadmium exposure, but more studies are needed that examine effects across multiple developmental stages.

It is not known whether effects of prenatal exposure on growth persist into adolescence or how concurrent adolescent cadmium exposure may impact this relationship. Focusing on adolescent adiposity outcomes is important given the dynamic physiologic changes occurring during the pubertal transition, including increases in weight and height velocity, accumulation and changes in lean and fat mass, and reproductive initiation and subsequent secretion of sex steroids (15, 16). Body composition changes—increase in total body mass and its distribution—are related to pubertal development and vary substantially by sex (17, 18). Cadmium exposure is associated with decreases in female and male sex hormones and a delay in pubertal development (19–22). Given the role of cadmium as an endocrine disrupting chemical, both *in utero* and peripubertal exposures may have significant effects on adiposity in adolescence (23, 24).

This study considers whether prenatal and concurrent cadmium exposures are associated with height, weight, BMI, and skinfold thickness at ages 8–15 years in a well-characterized birth cohort.

## Methods

### Study Population

The study population and methods for measuring cadmium exposure and anthropometric outcomes have been described in detail elsewhere (25, 26). Briefly, our study population consists of participants from the *Early Life Exposure in Mexico to Environmental Toxicants* (ELEMENT) project, wherein three sequentially enrolled cohorts of pregnant women were recruited between 1997 and 2004 from maternity hospitals in Mexico City serving low- to moderate-income populations. During their third trimester—mean gestation week 34 (standard deviation (SD) 2.3, range 25.1–40.4)—pregnant women provided a urine sample, anthropometry, and completed interview-based questionnaires. Between 2008 and 2011, a subset of child participants, who were now 8–15 years of age, were re-contacted based on availability of stored cord blood and maternal urine. At the time of re-recruitment, urine samples, anthropometry, and interview-based questionnaires were collected for children and adolescents (hereafter referred to only as “adolescents”).

The ethics and research committees of the Mexico National Institute of Public Health and the University of Michigan approved all research protocols, and all participants provided informed consent prior to enrollment.

### Cadmium Exposures

Urinary cadmium (UCd) was measured in 214 maternal third trimester (fasting spot) and 250 adolescent (arbitrary spot) urines. Urine was collected in sterile cups and samples were aliquot an hour after collection, frozen at −20 degrees Fahrenheit, and then moved to −70 degree Fahrenheit freezer for storage.

UCd was determined as previously described (27, 28). Briefly, 0.5 mL of sample was digested with an equal volume of concentrated nitric acid overnight in a fume hood. Milli-Q water was then added to the digest to bring the acid content to 2% (28). The digest was analyzed for Cd using an Inductively Coupled Plasma Mass Spectrometer (ICPMS; Varian 820MS). Potential elemental interferences were minimized via the ICPMS' collision reaction interface. The analytical accuracy was determined using a urinary reference material obtained from the Institut National de Santé Publique du Québec (QMEQAS10U-04) that has certified values for Cd. Every batch of 20 samples included a reference material and in total 25 measures were taken. The mean (SD) concentration of Cd measured in the reference material was 1.7 (0.1) μg/L compared to the actual certified value of 2.1 μg/L thus resulting in an accuracy of 82%. The analytical precision of urinary Cd was determined by creating a pool of urine that was run in each batch so that inter-day variability could be assessed. Every batch of 20 samples included a pooled sample and in total 25 measures were taken. The mean (SD) concentration of Cd measured in the pool was 0.13 (0.02) μg/L, providing a coefficient of variation (or precision) of 16%. In addition, each batch run contained procedural blanks that yielded a value of 0.01 (0.01) μg/L. The analytical detection limit was set as three times the standard deviation of the mean blank value, and was set at 0.04 μg/L; 190 (99%) of maternal and 215 (95%) of adolescent samples were greater than or equal to the limit of detection (LOD). For participants with UCd concentrations below the LOD, we retained UCd values between zero and the LOD.

UCd was corrected for specific gravity (SG) using the following equation: P_c_ = P[(SG_p_-1)/(SG_i_-1)] where P_c_ is SG-corrected UCd (μg/L), P is the measured UCd, SG_p_ is the population-specific median urinary specific gravity (maternal = 1.013, adolescent = 1.018), and SG_i_ is the individual's urinary specific gravity. Specific gravity (SG) was measured using a handheld digital refractometer (Atago Co., Ltd., Tokyo, Japan).

### Anthropometry

Trained study personnel used standard protocols to measure adolescents' anthropometry including waist circumference (cm), subscapular, suprailiac, and triceps skinfold thickness (mm), height (cm), and weight (kg). Subcutaneous subscapular and suprailiac skinfolds provide measures of truncal fat distribution and triceps skinfolds, obtained in the upper arm, reflect peripheral fat distribution. Skinfolds were measured with a calibrated caliper (Lange, Beta Technology) to the nearest 0.1 mm. Height and weight were obtained using professional scales (PAME, Puebla, Puebla) to the nearest 0.1 cm and 0.1 kg, respectively. Body mass index (BMI), defined as weight (kg) divided by height (m) squared $\left(\frac{kg}{m^{2}}\right)$, was calculated. Adolescent BMI z-scores were defined as BMI for age and sex, using the World Health Organization's reference standard (29).

### Covariates

Covariates included child age and sex and maternal BMI. Household socioeconomic (SES) level was measured with a 13-item score used in international settings to classify household assets (30). We coded maternal smoking history as never- or ever-smoker. Among ever-smokers, two percent reported smoking during pregnancy. Among mothers who reported a positive history of smoking, 70 percent also reported someone else smoking inside the home, including spouse, father, mother, and others. Thus, adolescents in our sample with a maternal history of smoking were potentially exposed from smokers in the home including mothers who often relapse by their child's first birthday (31, 32).

### Lead Exposure

Maternal bone (patella) lead levels were assessed at 1 month postpartum using *in vivo* K-X-ray fluorescence to describe cumulative *in utero* lead exposure (26, 33). Bone lead measures were available for a subset of the sample (*n* = 164). The distributions of cadmium exposure and anthropometric outcomes did not differ between those with and without lead measurements.

### Dietary Intake

Diets of pregnant women were assessed during the third trimester using an interview-administered semi-quantitative food frequency questionnaire (FFQ) designed to allow recall of dietary intake over the previous month (34). The questionnaire was translated and validated for use in the Mexican Spanish-speaking adult women of reproductive age; the list of about 100 foods was built from items that proved most representative of local consumption under the 1983 Dietary Survey of the Mexican National Institute of Nutrition (35, 36).

The energy content of each item in the FFQ (for a standard portion size: one natural unit, cup, slice, piece, etc.) was obtained from food composition tables supplied by two sources (Instituto Nacional de Salud Pública 2002): (1) the United States Department of Agriculture (USDA) and (2) the Mexican National Institute of Nutrition and Medical Sciences Salvador Zubirán (37). Frequency values, ranging from never to six or more times a day, were multiplied by the energy amount in each food item and then summed over all food sources to estimate the energy equivalent amount per day.

### Statistical Analysis

All statistical analyses were conducted using SAS version 9.2 (SAS Institute, Cary, NC, USA). The analytic dataset was limited to 185 adolescents, based on complete information of both prenatal and concurrent cadmium exposure measure and other covariates, including adolescent age and sex, household socioeconomic level, and maternal smoking history. No statistically significant difference was detected in mean cadmium exposures or anthropometry between the analyses of data with complete cases vs. data with all available cases.

Univariate statistics characterized distributions of cadmium exposure and anthropometric outcome variables. Variables with non-normal distributions were ln-transformed. Both prenatal and concurrent cadmium distributions were non-normal right-skewed; ln-transformed cadmium concentrations provided bell-shaped distributions and were used in analyses. Anthropometric outcomes that were ln-transformed include height, weight, waist circumference (WC) and subscapular, suprailiac, and triceps skinfold thicknesses (SFT); BMI z-score was not ln-transformed. Through the residual analysis, the appropriateness of such transformation was confirmed by the quantile-quantile normal plot. Wilcoxon signed-rank sum test determined the difference in median values by sex.

Bivariate correlation coefficients were used to identify covariates marginally associated with exposure and outcome. Potential covariates included birth cohort, adolescent age and sex, SES level, and maternal BMI and smoking history. Maternal bone lead and dietary intake were included in linear regression models as confounders.

**Figure 1:**
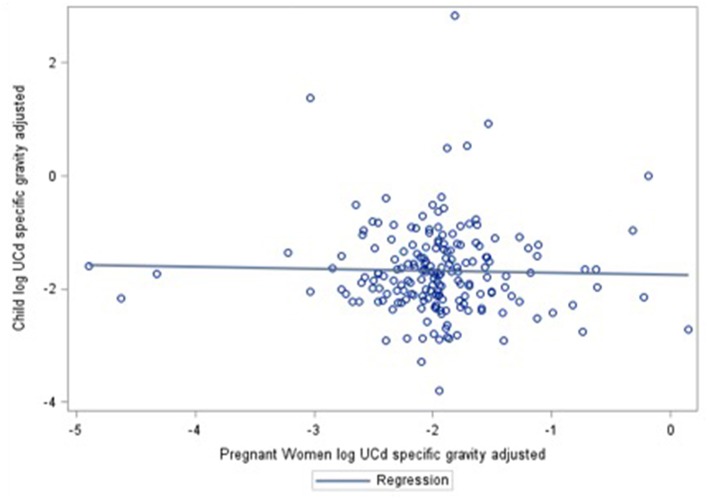
Correlation between prenatal and concurrent UCd concentrations.

Multiple linear regression was used to quantify the relationship of prenatal or concurrent cadmium exposure with anthropometric outcomes, unadjusted and adjusting for covariates. Each outcome was assessed individually. These relationships were quantified within the entire analytic sample and for prenatal and concurrent cadmium exposure, in separate analyses stratified by sex. We determined correlation between maternal-child cadmium measures (Figure 1) and estimated multiple linear regression of both prenatal and concurrent exposure on anthropometric outcomes. Results were back-transformed for interpretability and are reported as percent change in anthropometric outcome per inter-quartile range (IQR) increase in cadmium exposure. An interaction between maternal smoking history and cadmium exposure was not significant in linear regression models.

Confounding by maternal lead levels and dietary intake were assessed on the association of prenatal cadmium with anthropometry. Maternal lead exposure was correlated with prenatal cadmium exposure (*r* = 0.17, *p* = 0.028) and previous studies in this cohort demonstrate relationships of prenatal lead with childhood growth (26, 33, 38). Maternal fruit and vegetable intake was positively associated with urinary cadmium in pregnant women in our population, as previously published (25) and literature suggests that maternal diet is related to anthropometry in childhood (39).

Robust multiple linear regression was used to obtain stable results in the presence of substantial outliers in the UCd data. We used the classical multiple M-estimation procedure that combines high breakdown value estimation (Least Trimmed Squares) and conventional Huber's M estimation (40) to produce robust estimation and inference for association. Robust multiple linear regression models for prenatal cadmium associations with adiposity resulted in significant inverse relationships similar to multiple linear regression results reported.

## Results

The study population comprised 87 males and 98 females (median age 10 years, SD 1.5) residing in Mexico City. Data for the median and variation of cadmium exposures and anthropometry can be found in Table 1, overall and stratified by sex. Pregnant women and adolescents had median urinary cadmium concentrations of 0.19 μg/L (IQR 0.12–0.27 μg/L) and 0.14 μg/L (IQR 0.11–0.18 μg/L), respectively. Differences by sex were detected in skinfold thicknesses, with females having higher values reflecting expected shifts in fat distribution observed during peripuberty.

**Table 1:** Characteristics of adolescents and their mothers^a^.

	**Overall (*n* = 185)**			**Males (*n* = 87)**			**Females (*n* = 98)**			
	**Median**	**IQR**		**Median**	**IQR**		**Median**	**IQR**		***p*-value^b^**
**CADMIUM EXPOSURE**										
Prenatal UCd (μg/L)	0.19	0.12	0.27	0.16	0.11	0.23	0.19	0.12	0.31	0.67
Concurrent UCd (μg/L)	0.14	0.11	0.18	0.13	0.11	0.17	0.14	0.11	0.19	0.72
**ANTHROPOMETRY**										
Height (cm)	137	130	143	135	130	141	138	130	145	0.46
Weight (kg)	35.0	29.6	42.4	34.5	28.4	41.0	36.3	30.5	45.0	0.28
BMI z-score	1.0	−0.1	1.7	1.0	−0.1	1.8	1.0	0.0	1.6	0.95
WC (cm)	68.5	63.1	76.5	67.6	60.8	75.0	69.5	64.4	79.3	0.13
Subscapular SFT (mm)	10.5	7.0	15.5	9.3	6.0	13.5	11.5	8.0	18.0	<0.01
Suprailiac SFT (mm)	14.5	9.0	22.0	12.3	7.0	20.0	16.5	10.5	24.5	0.01
Triceps SFT (mm)	15.5	12.0	19.5	14.0	10.5	18.0	16.0	14.0	21.0	<0.01
**ADOLESCENT COVARIATES**										
Age (years)	9.9	8.8	10.7	9.8	8.8	10.7	9.9	8.8	10.7	0.80
Household SES level	8.0	6.0	10.5	8.0	6.0	10.5	8.0	6.0	10.5	0.90
**MATERNAL COVARIATES^c^**										
BMI	29.2	27.0	32.0	28.7	26.1	32.0	29.9	27.4	32.0	0.35
Bone Pb (μg/g)	7.7	1.4	13.8	5.9	1.5	13.7	8.6	1.3	14.3	0.52
Fruit intake (g/d)	46	39	57	46	39	57	46	39	57	0.88
Vegetable intake (g/d)	34	28	39	34	29	39	34	28	39	0.65
Energy intake (kcal/d)	1, 861	1, 536	2199	1, 927	1, 667	2, 203	1, 791	1, 484	2, 147	0.06

a *92 (50%) have a maternal history of smoking; 46 (53%) males and N = 46 (47%) females*.

b *Wilcoxon signed-rank sum test for difference in median values by sex*.

c *Maternal covariates were measured during the third trimester except bone lead, measured at 1 month postpartum*.

The results shown in Table 2 are obtained from separate models estimating the association of prenatal or concurrent cadmium exposure with adolescent anthropometry. Adjusted linear regression models showed negative relationships between prenatal cadmium exposure and adolescent anthropometry: height (*p* = 0.36), weight (*p* = 0.01), BMI z-scores (*p* = 0.01), waist circumference (*p* = 0.01), and subscapular (*p* = 0.01), suprailiac (*p* = 0.02), and triceps (*p* < 0.01) skinfold thickness (Table 2). A prenatal Cd exposure increase from the 25th to the 75th percentile (0.12–0.27 μg/L) was associated with a percent decrease in waist circumference (−3%) and subscapular, suprailiac, and triceps skinfold thickness (−11, −11, −8%, respectively); indicating higher prenatal cadmium exposure was associated with a decrease in both indicators of subcutaneous as well as abdominal fat. An IQR increase in prenatal Cd exposure was associated with a 27% decrease in BMI z-score, equivalent to a change of −0.27 in BMI z-score. Prenatal cadmium was negatively associated with weight. When stratified by sex, these relationships remained significant in girls but not boys (Table 3).

**Table 2:** Percent change in anthropometric outcomes per IQR increase in prenatal or concurrent UCd.

	**Prenatal UCd**				**Concurrent UCd**			
	**%Δ**	**95% CI**		***p*-value**	**%Δ**	**95% CI**		***p*-value**
Height	−0.32	−1.01	0.38	0.36	0.22	−0.24	0.68	0.35
Weight	−4.51	−7.89	−0.99	0.01	−0.68	−3.06	1.76	0.58
BMI z-score	−26.89	−46.31	−7.48	0.01	−7.93	−20.99	5.12	0.23
WC	−3.03	−5.20	−0.82	0.01	−0.46	−1.96	1.07	0.55
Subscapular SFT	−11.18	−18.24	−3.51	0.01	−2.48	−7.78	3.12	0.38
Suprailiac SFT	−10.74	−18.83	−1.84	0.02	−2.92	−8.91	3.46	0.36
Triceps SFT	−8.10	−13.31	−2.57	<0.01	−1.87	−5.66	2.08	0.35

*Adjusted for cohort, SES level, adolescent age and sex, and maternal BMI and smoking history*.

**Table 3:** Percent change in anthropometric outcomes per IQR increase in prenatal UCd stratified by sex.

**Outcome**		**%Δ**	**95% CI**		***p*-value**
Height	Overall	−0.32	−1.01	0.38	0.36
	Boys	−0.37	−1.32	0.58	0.44
	Girls	−0.40	−1.46	0.68	0.46
Weight	Overall	−4.51	−7.89	−0.99	0.01
	Boys	−2.22	−6.97	2.77	0.37
	Girls	−4.54	−7.93	−1.03	0.01
BMI z-score	Overall	−26.89	−46.31	−7.48	0.01
	Boys	−8.88	−37.79	20.03	0.54
	Girls	−39.75	−66.86	−12.64	<0.01
WC	Overall	−3.03	−5.20	−0.82	0.01
	Boys	−1.15	−4.32	2.12	0.48
	Girls	−4.61	−7.68	−1.43	0.01
Subscapular SFT	Overall	−11.18	−18.24	−3.51	0.01
	Boys	−6.54	−17.99	6.51	0.31
	Girls	−14.76	−23.80	−4.65	0.01
Suprailiac SFT	Overall	−10.74	−18.83	−1.84	0.02
	Boys	−6.54	−19.60	8.63	0.37
	Girls	−13.15	−23.53	−1.34	0.03
Triceps SFT	Overall	−8.10	−13.31	−2.57	<0.01
	Boys	−7.16	−15.71	2.27	0.13
	Girls	−8.85	−15.59	−1.57	0.02

*Adjusted for cohort, SES level, adolescent age, sex, and maternal BMI and smoking history*.

Adjusted linear regression models suggested negative relationships between concurrent cadmium exposure and adolescent anthropometry, although none were statistically significant (Table 2).

Supplemental Table 1 shows results obtained from additional sets of covariates included in the linear regression models. No correlation between cadmium exposure measures was found (*r* = −0.02, *p* = 0.75), which eliminates the potential collinearity issue when both are included in one model. Inclusion of both exposures in a single model to account for possible shared variance strengthened effect estimates by <10 percent and did not alter significance. Models with maternal lead and dietary intake did not substantially change effect estimates and confidence intervals overlap. Such a comparison of models exhibits the sensitivity or robustness of a chosen model on examining the relationship of prenatal UCd with anthropometric outcomes in adolescence.

## Discussion

We considered the independent associations of cadmium exposure in two time periods with adolescent anthropometry and found that prenatal but not concurrent cadmium exposure was significantly inversely associated with BMI z-score and measures of fat distribution, e.g., waist circumference and skinfolds. After stratifying by sex, we showed that these relationships were significant in females but not males. This is the first study to test whether the effects of prenatal cadmium exposure persist into adolescence, accounting for concurrent exposure.

Other published results are consistent with an inverse relationship between prenatal cadmium exposure and anthropometric outcomes in younger children (6–9, 41). A prospective study among Flemish children found inverse associations between prenatal cadmium exposure and measures of abdominal (WC) and subcutaneous fat (sum of subscapular, suprailiac, triceps, and biceps skinfold thickness) in females but not males 7–9 years of age. Our study similarly found statistically significant associations in females. However, our study differs from this and other reports by following children into adolescence and accounting for concurrent cadmium exposure.

Our study also has the advantage of using maternal urinary instead of cord blood cadmium to assess prenatal exposure. While blood and urinary cadmium are correlated, they may measure different aspects of exposure, dose and body burden. Blood cadmium is a measure of much more recent exposure (3–4 months) than that of urinary cadmium (10–30 years) (3). Additionally, placental transport of cadmium is limited (8, 42, 43), so offspring cord blood cadmium characterizes only direct recent exposure to the fetus, whereas maternal urinary cadmium may be more representative of maternal health throughout pregnancy (7, 8, 44). Evidence from animal studies suggest that fetuses exposed indirectly to Cd via maternal injection are more at risk than those directly exposed, possibly due to placental accumulation of Cd that results in trophoblastic damage (45), leading to decrease *in utero*-placental blood flow, and then a decrease in nutrient and oxygen transport (24). Use of maternal urinary cadmium to assess prenatal exposure could also be more indicative of effects on fetal epigenetic programming. A recent study found differences in epigenetic DNA methylation among infants and children at 4.5 years according to maternal cadmium exposure (46), with changes in methylation sites related to lower birth weight.

The only prior longitudinal study assessing cadmium exposure in multiple time periods did not find an independent association of prenatal cadmium exposure with growth of children in rural Bangladesh. After adjusting for prenatal and concurrent cadmium exposure and covariates (including exposure to lead), only concurrent childhood exposure was inversely associated with height and weight at 5 years (10). The inconsistency with our findings could be due to lower exposure levels in our sample; mean maternal urinary cadmium was 0.35 μg/L in our population compared with 0.65 μg/L in mothers of children in rural Bangladesh. Effects on growth at low levels of cadmium exposure previously have been documented in the literature with stronger associations observed for exposures below the median compared with those above the median (10, 12). Gardner et al. (10) did not assess measures of adiposity, e.g., BMI, WC and skinfold thicknesses among Bangladeshi children. Finally, these null findings were in children at 5 years of age whereas we examined the relationship of *in utero* cadmium exposure to anthropometry in adolescence, when measures of adiposity can change rapidly among boys and girls during puberty. We found a suggestive inverse relationship between concurrent cadmium exposure and adiposity. Previous cross-sectional studies in adolescents also have demonstrated negative relationships between concurrent cadmium exposure and weight, height, BMI, and WC (11, 12).

Cadmium may disrupt fat distribution through its endocrine properties, which may underlie the sex-specific results. Evidence in humans and animal studies suggests that cadmium disrupts steroidogenesis, particularly in the placenta, and acts as an endocrine-disrupting chemical, capable of the stimulation or inhibition of steroid hormonal activity (endogenous estradiol) (23, 24, 47). In laboratory studies, cadmium was observed to interact with both estrogen and androgen receptors and to activate the estrogen receptor alpha (48–50). The receptor-mediated mechanism of action may explain cadmium's pronounced effects at low-levels of exposure (12). In view of the stimulation of lipid mobilization and lipolysis known to be induced by sex hormones (51), this xenoestrogenic activity might contribute to the negative association with body fat observed for girls. In contrast, cadmium exposure in adolescent boys has been associated with lower circulating levels of estradiol and testosterone in the blood (22). Finally, in experimental animals, high doses of cadmium have been shown to lower plasma concentrations of insulin-like growth factor 1, a hormone critical for childhood growth, and disrupt pituitary levels (52–54).

Our study had the advantage of measuring confounding from lead and diet. When adjusting for cumulative *in utero* lead exposure, as measured by maternal patella bone lead, prenatal cadmium was still significantly inversely associated with adolescent weight, BMI z-score, WC, and SFT. Similarly, when Tian et al. adjusted for prenatal blood lead, prenatal cadmium remained significantly inversely associated with infant length and weight and child height at 4.5 years (47). Inclusion of maternal fruit and vegetable intake strengthened effects estimates, suggesting our first model may have underestimated the total effect of prenatal cadmium on adiposity. These results indicate that prenatal cadmium was independently associated with adolescent adiposity.

This study examined the relationship of cadmium exposure during two sensitive developmental periods with adolescent adiposity. Findings must be interpreted in light of several limitations. Use of a single urine sample to quantify UCd does not account for within-person variation and could attenuate associations with anthropometric outcomes (55). However, adjustment for urinary specific gravity can account for urine dilution. Our sample size was limited (*n* = 185) to participants with both measures of exposure, and stratification by sex limited our ability to consider interactions among covariates. Nevertheless, estimates of main effects reported here did not differ from models in which at least one time point for cadmium exposure was available (*n* = 192 prenatal and *n* = 223 concurrent) (results not shown). Finally, while we were able to account for maternal smoking history prior to pregnancy, more precise measures such as prenatal cotinine level and smoking habits of children may have provided more insight into relationships between different sources of cadmium exposure and growth outcomes.

## Conclusions

Prenatal cadmium exposure was negatively associated with measures of abdominal and peripheral adiposity in peripubertal girls, but not in boys. Prenatal cadmium remained an independent predictor of adiposity after adjusting for concurrent cadmium exposure and maternal lead exposure and dietary intake. These results emphasize the sex-dependent adiposity effects of cadmium exposure *in utero* that persist into adolescence.

## Author Contributions

MM and KP designed research. MM conducted research. MM and PS analyzed data. MM wrote the paper. AM-G and MT-R performed fieldwork. AC managed FFQ data. AJ provided global context. JC examined exposure data. MM and KP had primary responsibility for final content. All authors read and approved the final manuscript.

### Conflict of Interest Statement

The authors declare that the research was conducted in the absence of any commercial or financial relationships that could be construed as a potential conflict of interest.

## Abbreviations

SD: Standard deviation
IQR: Interquartile range
UCd: Urinary cadmium
BMI: Body mass index
WC, Waist circumference, SFT: Skinfold thickness.
