# Supplementary material for: Prenatal Cadmium Exposure Is Negatively Associated With Adiposity in Girls Not Boys During Adolescence
Source: Front Public Health. 2019 Apr 12;7:61. doi: 10.3389/fpubh.2019.00061 (PMC6473031; doi:10.3389/fpubh.2019.00061)
Supplement: Supplementary file 1 [file Table_1.DOCX]

**Supplemental Table 1. Percent change in anthropometry per IQR increase in prenatal UCd (95% CI) obtained for sets of covariates.^1^**

|  | Height | Weight |  |
| --- | --- | --- | --- |
| Model 1 | 0.63 (-0.87,2.15) | 1.02 (-3.29,5.53) |  |
| Model 2 | -0.32 (-1.01,0.38) | -4.51 (-7.89,-1.00) |  |
| Model 3 | -0.31 (-1.00,0.39) | -4.54 (-7.94,-1.03) |  |
| Model 4 | -0.27 (-1.01,0.48) | -4.18 (-7.78,-0.44) |  |
| Model 5 | -0.30 (-1.01,0.41) | -4.68 (-8.10,-1.12) |  |
|  |  |  |  |
|  | BMI z-score | WC |  |
| Model 1 | -24.74 (-43.53,-5.94) | -0.96 (-3.27,1.41) |  |
| Model 2 | -26.89 (-46.31,-7.48) | -3.03 (-5.20,-0.82) |  |
| Model 3 | -27.33 (-46.72,-7.94) | -3.06 (-5.23,-0.84) |  |
| Model 4 | -24.93 (-45.23,-4.63) | -2.82 (-5.10,-0.48) |  |
| Model 5 | -28.63 (-48.23,-9.03) | -3.23 (-5.41,-1.00) |  |
|  |  |  |  |
|  | Subscapular SFT | Suprailiac SFT | Triceps SFT |
| Model 1 | -5.06 (-12.81,3.38) | -6.50 (-14.87,2.70) | -3.99 (-9.55,1.92) |
| Model 2 | -11.18 (-18.24,-3.51) | -10.74 (-18.83,-1.84) | -8.10 (-13.31,-2.57) |
| Model 3 | -11.30 (-18.36,-3.64) | -10.88 (-18.96,-1.99) | -8.19 (-13.4,-2.67) |
| Model 4 | -9.97 (-17.53,-1.71) | -10.21 (-18.81,-0.71) | -7.70 (-13.27,-1.77) |
| Model 5 | -11.47 (-18.61,-3.70) | -11.56 (-19.66,-2.65) | -8.42 (-13.68,-2.84) |

^1^Model 1 – unadjusted

Model 2 – adjusted for cohort, SES, adolescent age and sex, maternal BMI and smoking history

Model 3 – adjusted additionally for concurrent Cd

Model 4 – adjusted additionally for maternal Pb

Model 5 – adjusted additionally for maternal intake of fruit and vegetables (energy adjusted).
